# A review on recent advances on nobiletin in central and peripheral nervous system diseases

**DOI:** 10.1186/s40001-023-01450-7

**Published:** 2023-11-06

**Authors:** Yueshan Pang, Juan Xiong, You Wu, Weijun Ding

**Affiliations:** 1 Nanchong Central Hospital, The Second Clinical Medical College of North Sichuan Medical College, Nanchong, 637000 China; 2 Department of Fundamental Medicine, Chengdu University of Traditional Chinese Medicine, Chengdu, 611130 China

**Keywords:** Nobiletin, Polymethoxyflavones, Central nervous system (CNS), Enteric nervous system (ENS), Neuroprotection

## Abstract

In recent years, the role of nobiletin in neuronal disorders has received extensive attention. However, the study of nobiletin in the peripheral nervous system is limited. Nobiletin, as a compound with high fat solubility, high bioavailability and low toxicity, has been extensively studied. Accumulating scientific evidence has shown that nobiletin has a variety of biological functions in the nervous system, such as inhibiting the expression of inflammatory factors, reducing the neurotoxic response, improving the antioxidant capacity, promoting the survival of nerve cells, promoting axon growth, reducing blood‒brain barrier permeability, reducing brain oedema, promoting cAMP response element binding protein expression, improving memory, and promoting mild depolarization of nerve cell mitochondria to improve antioxidative stress capacity. Accumulating studies have shown that nobiletin also protects enteric nervous system, spinal cord and sciatic nerve. To explore the new therapeutic potential of nobiletin in the nervous system, recent and relevant research progress is reviewed in this article. This will provide a new research idea for nobiletin in the nervous system.

## Background

The advantage of natural products is that they already exist in the human diet and can avoid the adverse reactions caused by some synthetic drugs. Citrus peel, as a byproduct of fruit and a traditional Chinese herb, has been widely used since ancient times. Nobiletin, a natural extract of citrus peel, has been widely studied in nervous system diseases, such as Alzheimer’s disease (AD) [12], depression [32], stroke [60, 62], amyotrophic lateral sclerosis [18], and even enteric nervous system disease [16, 46]. However, its specific molecular mechanisms are still unclear. While there are a few reviews on the effects of citrus peel or nobiletin on the nervous system, there is still no systematic description of the absorption, distribution, effect and mechanisms of nobiletin in the central and peripheral nervous systems. Therefore, we reviewed nobiletin in in the context of central and peripheral nervous system diseases. Such work may provide a state-of-the-art reference and prospective intervention strategy for central and peripheral nervous disorders.

## Search strategy

**Fig. 1:**
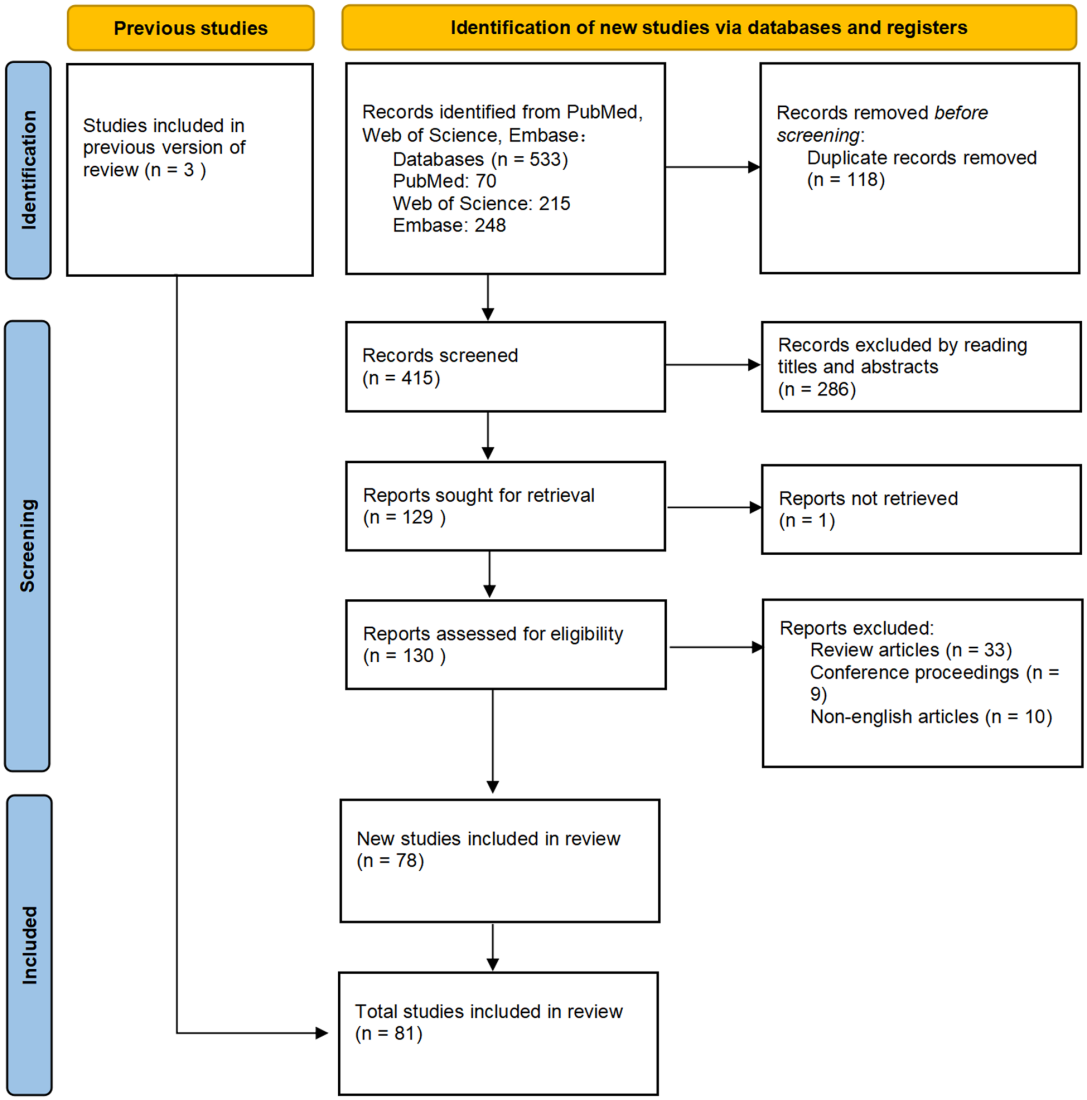
The flow of the literature search. A total of 536 articles were retrieved. Among the retrieved articles, 455 articles were excluded for the following reasons: (1) articles belonging to meeting abstracts, reviews, letters, editorials, expert opinions, and case reports; (2) literature written in a language other than English; and (3) content not related to keywords. A total of 81 articles were ultimately included in this review

We searched relevant studies by using databases including PubMed, Embase, and Web of Science restricted to April 24, 2023. The Medical Subject Headings and Embase Subject Headings resources were used for selecting the keywords and free words. Our search strategies included the following terms: 1# the keywords “nobiletin”, “methoxyflavones”, “hexamethoxyflavoneor”, “polymethoxylated flavones”, “citrus folium”, “citrus peel” and “tangerine peel” were combined with their free words, and the Boolean logic word “OR” was used; 2# the keywords “nervous system disorders”, “enteric nervous system”, “central nervous system”, “peripheral nervous system”, “brain diseases” and “brain” were combined with their free words, and the Boolean logic word “OR” was used. The Boolean logic word “and” was used between search strategies #1 and #2. Furthermore, a manual search of reference lists in the selected articles was performed. Four additional studies were included in the original article search. Yueshan Pang and Juan Xiong independently reviewed all the identified studies and selected full papers for analysis according to the inclusion and exclusion criteria. The inclusion criteria for selecting the articles were as follows: research content related to keywords or free words. The exclusion criteria were as follows: (1) articles belonging to meeting abstracts, reviews, letters, editorials, expert opinions, and case reports; (2) literature written in a language other than English; and (3) content not related to keywords. Any discrepancies between the two investigators were determined independently by the third investigator (You Wu). The study process is shown in Fig. 1. The mechanism maps were drawn using ScienceSlides.

## Absorption and distribution patterns of nobiletin

The biotransformation of nobiletin in the intestine. There are three main pathways for the entry of nobiletin into the body: oral administration, intravenous injection and intraperitoneal injection. After oral administration, nobiletin is metabolized by intestinal microbiota into more active products [29, 79]. The intestinal microbiota of healthy individuals can cause demethylation of nobiletin [7, 29]. After demethylation, flavonoids with stronger solubility and biological activity can be produced [7, 14].

*The metabolism and absorption of nobiletin*. Nobiletin has a higher intestinal absorption rate than other flavonoids [43]. A permeability test with an artificial membrane showed that the permeability of nobiletin was very high at both pH 4.0 and pH 7.0 [55], which indicates that nobiletin may be absorbed in the entire intestinal tract. There is a first-pass effect after nobiletin is absorbed by the intestinal tract [28]. Liver P450 reductases such as CYP2C11, CYP2C12, CYP2D1, CYP3A1 and CYP3A2 are responsible for demethylation at the 6-, 7-, 3- and 4-positions in rings A and B, whereas CYP1A1 and CYP1A2 preferentially catalyse demethylation at the 3- and 4-positions in ring B [28]. Studies have shown that almost all nobiletin undergoes demethylation after oral administration, and only a small amount of nobiletin itself was identified in the urinary metabolites [34, 35, 72]. 4'-Demethylated nobiletin is the main metabolite of nobiletin, and 24-h urine excretion only accounts for 13.19 ± 1.43% of the total amount of oral nobiletin [72]. Moreover, this secondary metabolite of nobiletin has a stronger anti-inflammatory effect [34]. Nobiletin can bind to serum proteins but does not affect the overall conformation of serum proteins [73], which reduces the toxicological properties and increases bioavailability.

*The distribution of nobiletin in vivo*. Nobiletin can be detected in brain tissues 5 min after intravenous injection [4, 50, 69]. After oral administration of nobiletin (50 mg/kg), the concentrations in the blood and brain peaked at 1.78 μg/mL and 4.20 μg/mL, respectively [55]. The half-lives of nobiletin in the plasma and brain are 1.80 h and 11.42 h, respectively [55]. The concentration of nobiletin in the brain is higher when taken intravenously than when taken orally [4], which means that intravenous administration is the preferred method for central nervous system diseases. Nobiletin has high lipid solubility and permeability in the blood‒brain barrier [54]. The preparation of nobiletin into nanoscale amorphous dispersed solids can further increase its concentration in the brain and increase its bioavailability [44], which is conducive to the treatment of central nervous system diseases. In brief, these characteristics of nobiletin make it an ideal candidate for drug research and development.

## Effect and mechanisms of nobiletin in the CNS

### AD model

Molecular docking studies have also shown that nobiletin can stably bind to beta-site amyloid precursor protein cleaving enzyme 1 (BACE1), inhibit BACE1 activity, and suppress Aβ protein production [40, 76]. Nobiletin can promote Aβ degradation by activating neprilysin [10, 27, 45]. In addition, nobiletin suppresses inflammation via the TLR4/NF-kB/Nrf2 [12] and JNK [75] pathways and suppresses iNOS and COX-2 expression and neuronal apoptosis in an Aβ-induced AD model [75].

After treatment with nobiletin, the expression of HMGB1 and pyroptosis-related proteins (nucleotide-binding oligomerization domain-like receptor 3, ASC, cleaved caspase-1, and N-terminal fragment of gasdermin D) was inhibited in an AD model [8]. Nobiletin can significantly inhibit AChE activity [30], increase ChAT expression and alleviate cognitive dysfunction [23, 41].

**Fig. 2:**
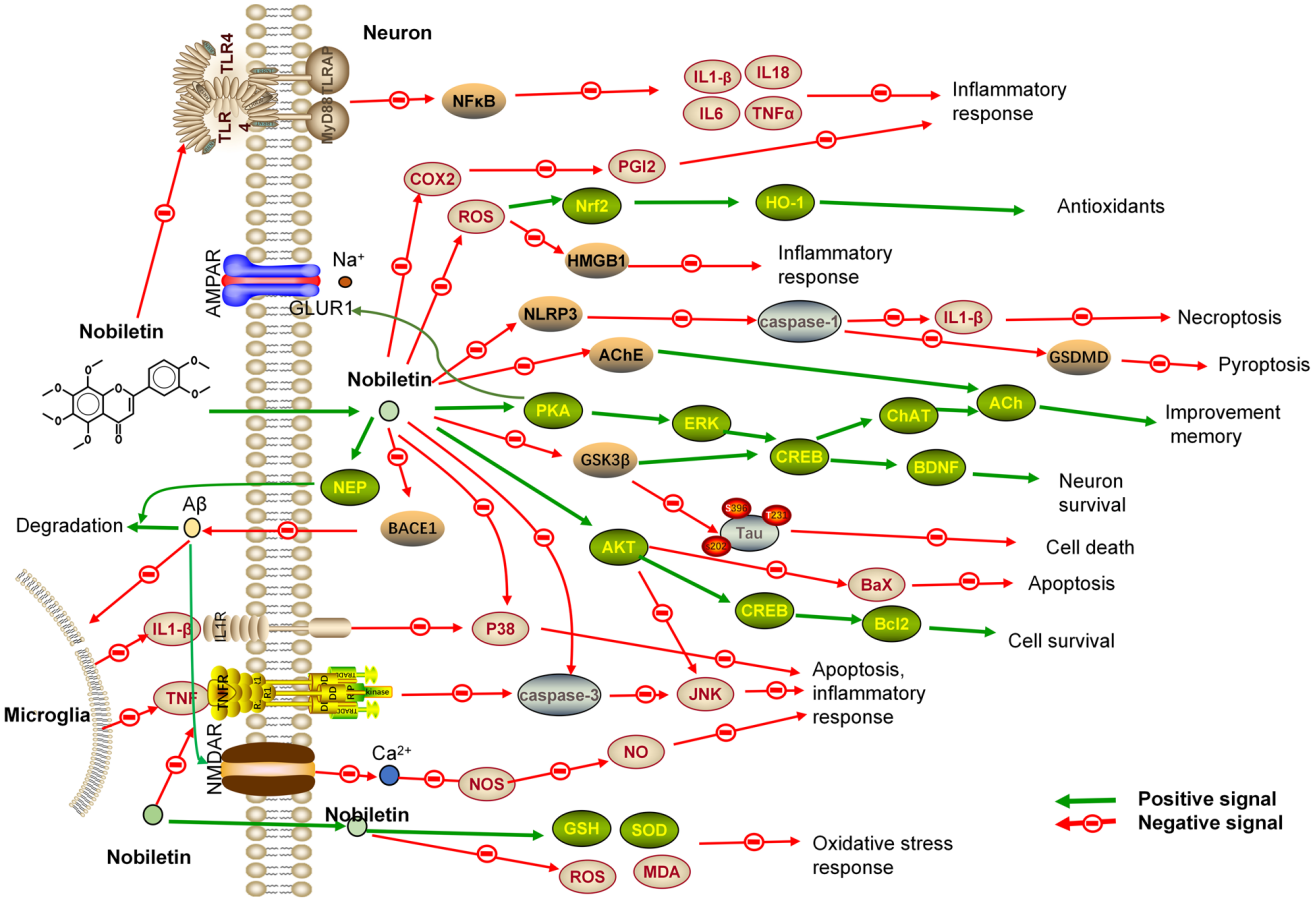
The mechanisms of nobiletin. Nobiletin inhibits microglial activation and inflammatory factor production to reduce apoptosis in neuronal cells. Nobiletin inhibits apoptosis, inflammation and oxidative stress action directly on the neuronal cells. Nobiletin also promotes neuronal cell survival and improves memory impairment

Pathways related to nobiletin in cognitive dysfunction. Nobiletin protects hippocampal neurons through the cAMP/PKA/ERK/CREB signalling pathways and improves learning and memory ability [11, 24, 38, 39, 58]. Nobiletin and nerve growth factor act together to increase the phosphorylation level of ERK and activate CRE-dependent transcription [58]. Intraperitoneal injection of 10 or 50 mg/kg 4-demethylnobiletin can improve NMDA receptor dysfunction-induced cognitive dysfunction via the PKA/ERK/CREB pathway [2]. PKA is a key enzyme in the formation of memory. After treatment with nobiletin, the expression level of PKA and the phosphorylation level of the AMPA receptor subunit GluR1 were significantly increased, thereby regulating local postsynaptic potentials [37]. Isoflurane-induced cognitive disorder can also be improved by nobiletin by promoting the expression of Akt, Bax, p-CREB and BDNF in the brain [5]. The mechanisms of nobiletin are shown in Fig. 2, and the treatment-related information for nobiletin is described in Table 1.

**Table 1:** Treatment-related information of nobiletin

Dose and route of administration	Time	Model	Effects	References
10, 50 or 100 µM	/	Cell-derived AD model	BACE1 inhibitory	[76]
10 – 50 mg/kg (i.p.)	7 d	AD model mice	Soluble Aβ_1–40_↓ learning and memory impairment ↓	[40]
0, 10, 50, 30 or 100 µM	72 h	Cell-derived AD model (SK-N-SH cells)	Neprilysin activity ↑	[10]
3, 10 or 30 µM	24 h	Cell-derived AD or normal neurons model	Neprilysin mRNA ↑ Aβ_1–42_ ↓	[27]
10 mg/kg (i.p.)	4 m	AD model mice	Guanidine-soluble Aβ_1–40_ and Aβ_1–42_ ↓	[45]
1, 10, 25 or 50 µM	1 h	Cell-derived AD or normal neurons model	Oxidative stress ↓ Interleukin-1 ↓ Tumour necrosis factor-α↓ Nitric oxide (NO)↓ Prostaglandin E2↓ Cyclooxygenase-2↓ c-Jun N-terminal kinase and p38 ↓	[75]
25, 50 or 75 mg/kg (p.o.)	90 d	AD model mice	Apoptosis↓ IL-1β↓, TNF-α↓, and IL-18 levels ↓ HMGB-1↓, NLRP3↓, ASC↓ Cleaved Caspase-1↓, GSDMD-N ↓	[8]
30 mg/kg (p.o.)	4 w	Aβ 1–42 injection mice model	AchE activity↑ Bax and cleaved caspase-3 ↓ Bcl-2 and Bcl-2/Bax ↑	[30]
50 mg/kg	11 d	AD model mice	ChAT ↑	[23]
30 or 100 μM	18 h	PC12D cells and hippocampal neurons	ChAT ↑, AchE ↑ Cholinergic neurodegeneration ↓	[41]
30 μM	5 h	PC12D cells	CRE-mediated transcriptional Activity ↑ ERK phosphorylation level ↑	[11]
25 mg/kg	7 d	AD Model mice	CRE-mediated transcriptional activity ↑	[24]
10 μM	/	PC12 cells	CRE-dependent transcriptional activity ↑ Erk phosphorylation ↑	[58]
10 or 50 mg/kg	7 d	AD Model mice	CREB phosphorylation ↑ Learning ability ↑	[38]
100 μM	8 h	PC12D cells	Neurite outgrowth ↑ Improve impaired memory ↑ CRE-dependent transcription ↑	[39]
10 or 50 mg/kg (i.p.)	7 d	AD model mice	PKA/ERK/CREB signalling ↑ learning impairment ↓	[2]
100 μM	15 min	PC12D cells	protein kinase A phosphorylation ↑ GluR1 receptor phosphorylation ↑	[37]
10 or 25 mg/kg (i.p.)	3 d	AD model mice	phosphorylated ‑Akt ↑ CREB ↑, BDNF ↑ Bax ↓	[5]
25 or 50 mg/kg (i.p.)	7 d	Stroke model mice	Calcium/calmodulin-dependent protein kinase II ↑ Microtubule-associated protein 2 ↑ Glutamate receptor 1 ↑	[67]
1 μM	48 h	Cell hypoxia model	Astrocytes activation↓ Nrf2 nuclear translocation ↑ HO-1 expression ↑ GFAP ↓ ROS and MDA ↓ Mitochondrial dysfunction ↓	[60]
10 or 25 mg/kg(i.p.)	3 d	Stroke model rat	Brain oedema ↓ Infarct volume ↓ p-Akt↑, CREB↑, BDNF↑, Bcl-2 ↑ Claudin-5 ↑	[78]
15 mg/kg (i.p.)	1 d	Stroke model rat	Infarct volume ↓ Brain oedema ↓ Neutrophil invasion ↓ Apoptotic ↓	[71]
10 or 25 mg/kg(i.p.)	3 d	Stroke model rat	Neurological deficits ↓ Brain oedema ↓ Infarct volume ↓ Nrf2 ↑, HO-1↑, SOD1↑, GSH ↑ NF-κB ↓, MMP-9↓, MDA ↓	[77]
100 or 200 mg/kg (p.o.)	9 d	Stroke model rat	TNF-a↓, IL-1β↓, IL-6↓, NO ↓ TLR4↓, NF-κB ↓	[80]
1, 10, 20 or 50 µM	24 h	Stroke cell model	Endoplasmic reticulum (ER) stress(ERS) -induced apoptosis ↓ Dehydrogenase ↓ Cellular viability ↑ PI3K/AKT pathway ↑	[36]
0,10,50 or 100 µM	24 h	LPS-stimulated BV-2 microglia	TNF-a, IL-1β ↓ NF-kB ↓, ERK ↓, p38 ↓, JNK phosphorylation ↓	[9]
25 or 100 μg/ml	24 h	LPS-stimulated microglia	NO↓, iNOS↓, NF-κB↓, MAPK phosphorylation ↓	[19]
3–10 μM	24 h	BV‐2 cells	IL‐1β↓	[17]
40 μM	20 h	LPS-stimulated BV-2 microglia	NO ↓, TNF-a ↓, IL‐1β↓, IL-6 ↓	[1]
100 mg/kg (p.o)	10 d	LPS intrahippocampal challenge	Memory deficit ↓ COX-2 ↓, IL-1β ↓, TNF-α ↓, and iNOS↓	[48]
100 or 200 mg/kg (p.o.)	6 w	LPS-induced neuroinflammation	iNOS↓, IL-6 ↓, JAK2↓, TNF↓, IL-1↓, and NF-κB↓ STAT3 phosphorylation ↓	[63]
6.25, 12.5, 25 or 50 μg/ml	24 h	LPS-stimulated BV-2 microglia	NO↓, iNOS↓, IL-6↓, JAK2↓, TNFα↓, IL-1β↓, and NF-κB ↓	[57]
10, 20 or 40 μM	24 h	H2O2-induced oxidative stress in astrocytes	Ose-regulated protein(GRP) 78 ↑, Cell death ↓ Endoplasmic reticulum (ER) stress lead ↓	[21]
30, 50, 100 or 200 μM	10 min	Glutamate-stimulated neurons	Calcium overload ↓ ROS ↓ Mitochondrial depolarization ↑	[31]
1, 10 or 30 µM	5 min	Neurons	ROS↓, apoptotic signalling↓, ATP production ↑, Neuronal viability↑, Nrf2↓, HO-1↓	[3]
10 mg/kg (i.p.)	9 d	Cisplatin-induced nerve injury	Peroxide↓, apoptotic↓	[25]
50 µM	96 h	Sodium arsenate-induced neural progenitor cells toxic	Neuronal degeneration ↓, Oedema ↓, caspase-3↓ BDNF ↑, G6PD activity ↑ Antioxidant ↑, Antiapoptotic ↑ Neuroprotective effects ↑	[42]

In summary, nobiletin can inhibit BACE1 activity, suppress the production of Aβ protein and ameliorate cognitive dysfunction in AD.

### Stroke model

Nobiletin improved stroke-induced learning and memory impairment by promoting the expression of Ca2 + /calmodulin-dependent protein kinase II (CaMKII), microtubule-associated protein 2 and glutamate receptor 1 and increasing ERK and CREB phosphorylation levels [67]. In an in vitro glial cell hypoxia model, nobiletin promoted Nrf2 nuclear translocation and haem oxygenase-1 (HO-1) expression and inhibited the hypoxia-induced oxidative stress response [60]. Nobiletin reduces the permeability of the blood‒brain barrier and cerebral oedema by increasing the expression of claudin-5 [78]. After intravenous injection of nobiletin, nobiletin rapidly accumulates in the infarcted area to protect against nerve damage by activating the Akt/CREB pathway [71, 78], increasing the expression of BDNF [30, 62, 71, 78], and reducing neutrophil infiltration and neuronal apoptosis [71]. Nobiletin reduces local inflammation after stroke by inhibiting NF-κB/MMP9 signalling [77]. Nobiletin also improves the antioxidant capacity after stroke by increasing the activity of SOD1 and GSH and reducing MDA content [77]. Nobiletin can reduce the expression of NO, IL-1β, IL-6, Bax, caspase-3 and TNF-α inflammatory cytokines [62, 80] and increase IL-10 expression through the TLR4/NF-κB and MAPK signalling pathways after stroke [62]. Nobiletin also promotes cell survival and reduces apoptosis by activating the Akt/mTOR/GSK-3β pathway in stroke [80]. In the oxygen–glucose deprivation–reperfusion model of PC12 cells, it was also confirmed that nobiletin activated PI3K/Akt and reversed endoplasmic reticulum (ER) stress (ERS)-induced apoptosis to reduce neuronal injury [36]. The treatment-related information of nobiletin is described in Table 1.

In brief, nobiletin inhibits inflammation and oxidative/nitrosative stress to protect neurons and facilitate neurological recovery in stroke.

### Parkinson’s disease (PD) model

PD is associated with the progressive degeneration of dopaminergic cells. However, increasing evidence suggests that PD may be associated with inflammation and a reduction in glial cell line-derived neurotrophic factor (GDNF) [22, 66]. Nobiletin (50 mg/kg, i.p.) can increase the CaMKII autophosphorylation level, increase the activity of tyrosine hydroxylase and reduce the loss of dopamine neurons to improve 1-methyl-4-phenyl-1,2,3,6-tetrahydropyridine-induced Parkinson-like symptoms [22, 66]. Nobiletin (1, 10 or 20 mg/kg, i.p.) also inhibits microglial activation, promotes GDNF expression, and reduces neurodegenerative lesions in PD [22]. Although the number of studies on nobiletin in PD is small, further research should be conducted in this area.

### Anxiodepressive model

A study has shown that citrus peel extract produced has a strong antidepressant effect [32]. The antidepressant effect of nobiletin (10 mg/kg, p.o.) is achieved through the serotonergic, dopaminergic and noradrenergic systems [74]. In addition, the antidepressant effects of nobiletin (20 or 40 mg/kg, p.o.) were also shown to increase the expression of BDNF, TrkB and synapsin I in the hippocampus [33]. Nobiletin can improve behavioural symptoms by reducing iNOS, IL-1β, IL-6, COX-2 and inflammasome expression through the AMPK pathway in a lipopolysaccharide-induced depression model [61]. Nobiletin (100 μM) also increases autophagy by increasing LC3-II and Beclin-1 expression through activating the AMPK pathway to improve LPS-induced depressive symptoms in a BV2 cell model [61]. In conclusion, the antianxiodepressive effect of nobiletin is associated with anti-inflammatory effects and improves the expression of nerve growth factor.

### Epilepsy model

Epilepsy is a complex disease that may be the result of increased excitability of neurons in several regions of the brain. Nobiletin (12.5, 25 or 50 mg/kg, p.o.) may inhibit neuronal apoptosis by increasing Bcl-2 and Bcl-xL expression and reducing caspase-3, Bad, and Bax expression in epilepsy models [68]. The BDNF–TrkB signalling pathway is critical for the development of epilepsy, and nobiletin also inhibits seizures by inhibiting the BDNF–TrkB pathway [68]. Nobiletin also reduces the incidence of seizures by reducing Glu/GABA levels [6, 68]. In addition, nobiletin can increase GSK-3β, mTORc-1, and mTORc-2 levels through the PI3K/Akt pathway to inhibit epileptic seizures [68]. In conclusion, the effect of nobiletin in epilepsy is associated with the apoptotic, BDNF-TrkB and PI3K/Akt signalling pathways.

### Disordered circadian clock model

In recent years, the function of PMFs in regulating the biological clock has gradually attracted attention. A clinical study of AD showed that nobiletin, which is enriched in orange peels, can improve abnormal mental behaviour. Midazolam can induce circadian rhythm disorders by reducing PER2 in the hippocampus; however, this can be alleviated by nobiletin (1 mg/kg, i.p.) through enhancing the amplitude of PER2 [13]. Another study also suggested that nobiletin (containing 0.1% diets) can significantly increase the temporal changes in the expression of Clock, Bmal1 and Npas2 [26, 64]. Nobiletin (1, 10, 30 or 50 mg/kg, i.p.) improved surgery-induced neurocognitive decline by preserving the expression of the clock genes Bmal and Rors [56]. A disordered central circadian clock is related to many diseases. In this review, nobiletin improved the disordered central circadian clock by regulating clock genes. It would be interesting to investigate this further.

### Brain tumour model

Although brain tumours rarely metastasize to distant sites, their diffuse and invasive growth in the brain usually directly affects the success of therapy. Therefore, finding new adjuvant therapy drugs has always been the eternal topic of antitumour therapy. Nobiletin (425 μM or 4 mg/ml) can inhibit the expression of MMP-2 and MMP-9 in glioma cells and reduce the motility, adhesion and invasion ability of glioma cells [49, 51]. The antitumour effect of nobiletin in the brain is associated with reducing adhesion and invasion of glioma cells, and more mechanistic studies deserve to be further explored.

### Neuroinflammation and neurotoxicity model

Nobiletin can suppress lipopolysaccharide-stimulated BV-2 glial cell activation by inhibiting the phosphorylation levels of ERK, p38 and JNK [9, 19] and NO, TNF-a, IL-1β, IL-6 and iNOS expression through NF-κB signalling [1, 9, 17, 48, 63]. Nobiletin also inhibits IL-1β, IL-6, and TNF-α expression by inhibiting the JAK2/STAT3 pathway [63].

After injection of nobiletin, the expression of GRP78 was increased, and endoplasmic reticulum stress and neurotoxicity were decreased in a 1-methyl-4-phenyl-1,2,3,6-tetrahydropyridine-induced neurotoxicity model [57]. Excessive glutamate has been considered an excitotoxic agent in neural cells, and glutamate receptors have become targets for the treatment of this neurotoxicity. Molecular docking studies showed that nobiletin could stably bind to glutamate receptors, indicating that nobiletin is a potential therapeutic drug for glutamate-induced neurotoxicity [21]. Nobiletin also increased mitochondrial K^+^ influx to mildly depolarize mitochondria and reduce mitochondrial calcium overload to protect nerves [31].

**Fig. 3:**
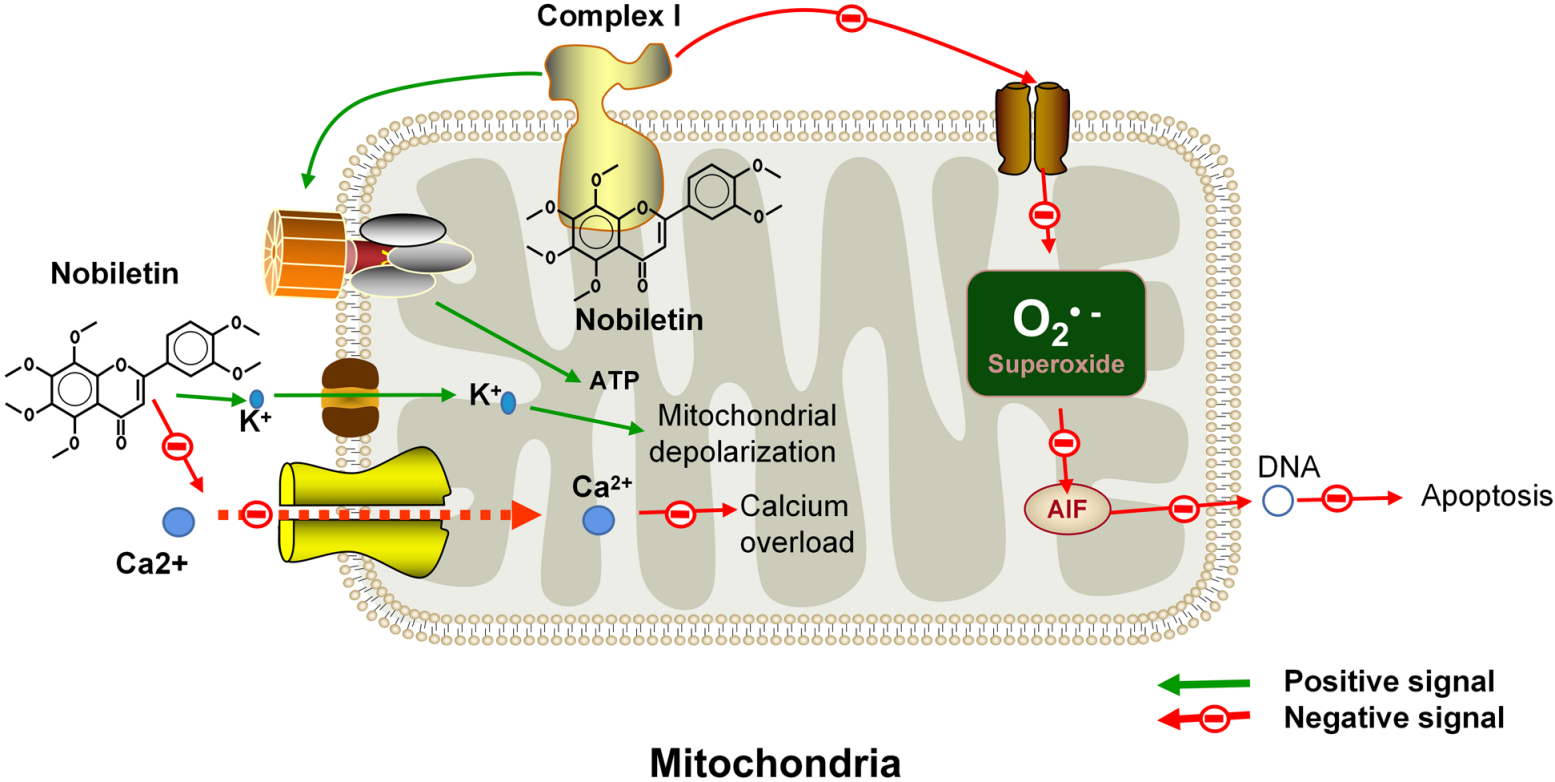
Mechanisms of nobiletin in mitochondria. Nobiletin inhibits mitochondrial calcium overload by blocking calcium influx. Nobiletin promotes mitochondrial depolarization by increasing potassium influx and ATP production. Nobiletin inhibits oxidative stress and apoptosis via complex I

There are two effects of nobiletin treatment on rotenone-induced neurotoxicity. When complex I is in the activated state and binds to nobiletin, complex I can be inhibited, thereby inhibiting oxidative stress defence and increasing cell viability; when complex I is in the inhibited state and binds to nobiletin, the brain mitochondrial oxygen consumption rate can be improved [3]. Through binding to the mitochondrial complex I subunit NDUFV1, nobiletin can decrease complex I activity and inhibit ROS production, thereby reducing mitochondrial apoptosis-inducing factor (AFI) nuclear translocation and suppressing nerve cell apoptosis [3]. Nobiletin can also promote ATP production, activate the mitochondrial α-ketoglutarate dehydrogenase complex, cause mild mitochondrial depolarization, and accelerate the phosphorylation of the matrix substrate level to protect nerves from neurotoxic effects [3, 53]. The neuroprotective mechanisms of nobiletin in mitochondria are shown in Fig. 3.

Nobiletin increased BDNF concentration and glucose-6-phosphate 1-dehydrogenase activity and reduced caspase-3 expression to protect against chemotherapeutic drug cisplatin-induced neurotoxicity [25]. Nobiletin has therapeutic effects on sodium arsenate-induced neurotoxicity [20]. Nobiletin can restore morphological damage (neurite damage), restore the levels of stress granule ras-GTPase-activating protein (SH3 domain)-binding protein and T cell-restricted intracellular antigen, and increase the expression of the neural marker proteins β tubulin, Nestin, and Pax6 [20]. Nobiletin also restores arsenic-induced mitochondrial membrane potential damage [20]. The treatment-related information of nobiletin is described in Table 1.

In summary, nobiletin reduces neuroinflammation and neurotoxicity. Nobiletin reduces neuroinflammation and is associated with suppressing microglial activation. Mitochondria is one of targets for nobiletin in neuroinflammation and neurotoxicity model and that deserves further investigation.

## Effect and mechanisms of nobiletin in the peripheral nervous system

### ENS

The enteric nervous system is known as the second brain. Nobiletin has a bidirectional regulatory effect on jejunal movement. Low doses of nobiletin (1.25–5.0 μM) showed a promoting effect, and high doses of nobiletin (10.0–40.0 μM) showed an inhibitory effect on normal contractility in the jejunum [65]. Nobiletin stimulates low contractility jejunum movement while inhibiting high contractility jejunum movement [15, 65]. The inhibitory effect of nobiletin on the jejunum is related to NO secretion by nitrergic neurons, and the contraction-promoting effect on the jejunum is related to Cajal cells [65]. The Ca^2+^ channel is another indispensable factor for the bidirectional regulatory effect of nobiletin [65]. Nobiletin can increase epinephrine-induced low [Ca2 +]i and reduce ach-induced high [Ca2 +]i status [65]. Nobiletin (100 μM) can significantly reduce the expression of LPS-induced inflammatory factors, such as TNF-α, NF-κB, COX-2 and iNOS, in RAW264.7 cells [15]. A recent study also suggested that nobiletin (100 or 200 mg/kg, p.o.) inhibited HFD-induced colon inflammation and improved colon motor function by increasing Trem2 expression [46]. The ENS has been a focus of research in recent years. The ENS has been implicated in the development of various diseases. This review suggests that nobiletin protects the ENS and regulates intestinal motor functions. Nobiletin in ENS is deserving of further research and development.

### Spinal cord diseases

Amyotrophic lateral sclerosis is a degenerative disease of motor neurons distributed in the cortex, brain stem, and spinal cord. Superoxide dismutase type 1 (SOD1) mutations lead to protein aggregation and reduced protein stability, which is associated with amyotrophic lateral sclerosis. Nobiletin (2 or 10 μM) significantly alleviates autoimmune encephalomyelitis symptoms by inhibiting Th17 cell differentiation and interleukin-17A production [42]. Nobiletin (25 or 50 mg/kg, i.p.) also significantly reduces the expression of TNF-α, IL-1β and IL-6 in the brain and spinal cord and increases the expression of IL-10, TGF-β and interferon γ to reduce the inflammatory response in the brain and spinal cord in encephalomyelitis [70]. In brief, nobiletin also suppresses neuroinflammation in the spinal cord.

### Other peripheral nerves

Sciatic nerve injury leads to sensory and motor impairment. Nobiletin can inhibit H_2_O_2_-induced sciatic nerve cell toxicity [52]. Nobiletin (50 or 100 μM) can significantly promote synaptic growth of sciatic nerve cells, which is associated with the BDNF-ERK1/2 and AKT pathways [52]. At present, the clinical treatment effect of sciatic nerve injury caused by long-term chronic compression is poor. However, a study has shown that the administration of nobiletin (30, 60, 120 mg/kg) for 8 days can significantly improve the pain of the long-term compressed sciatic nerve, which was associated with inhibition of the IRF5/P2X4R/BDNF signalling pathway in spinal microglia [81]. In conclusion, nobiletin had a protective effect on the sciatic nerve.

## Conclusions

The major effects of nobiletin on the nervous system are inhibiting inflammatory cytokine expression [1, 48, 63], reducing neurotoxicity [20, 21, 25], promoting the survival of nerve cells and axon outgrowth [52], reducing blood‒brain barrier permeability and cerebral oedema [36, 59, 60, 77], promoting mild depolarization of nerve cell mitochondria and increasing capacity against oxidative stress [3, 53]. The biological roles of nobiletin are inseparable from its basic mechanism. Nobiletin has two or more methoxy groups, has no glycosidic bonds, is more hydrophobic than hydroxyl compounds, and has a higher intestinal absorption rate than other flavonoids [43]. Nobiletin also has high permeability in the blood‒brain barrier due to its high lipophilicity [54]. The hydroxyl groups at the C-3 and C-4 positions of nobiletin are necessary for its antioxidative capacity and radical scavenging capacity [47]. Nobiletin has a native structure for scavenging free radicals, high lipophilicity and high bioavailability and is worthy of further development and research as a drug.

There are many studies of nobiletin in the CNS, and the effect of nobiletin on the peripheral nervous system has gradually attracted attention in recent years [15, 42, 46, 65, 70], especially in the ENS [46]. Many risk factors cause ENS damage. However, there are few effective drugs for ENS damage. The intestinal microbiota can cause nobiletin demethylation [29]. After demethylation, nobiletin has a stronger solubility and biological activity [14]. A study has shown that nobiletin protects against HFD-induced ENS damage [46]. However, further studies are necessary to deeply elucidate the mechanism of nobiletin in the ENS. After nobiletin treatment, whether there are also alterations in neuroinflammation, apoptosis and neurogenesis in the ENS is still unclear. Hence, this is an intriguing direction for future research. Therefore, we reviewed the effects and mechanisms of nobiletin in the nervous system to provide a state-of-the-art literature and prospective intervention strategy for nervous disorders by nobiletin.

## Data Availability

Not applicable.

## Abbreviations

CREB: CAMP response element binding protein
PMFs: Polymethoxy flavonoids
AD: Alzheimer’s disease
ENS: Enteric nervous system
CYP2C11: Cytochrome P450 2C11 precursor
CYP2C12: Cytochrome P450 2C12 precursor
CYP2D1: Cytochrome P450 2D1
CYP3A1: Cytochrome P450 3A1
CYP3A2: Cytochrome P450 3A2
CYP1A1: Cytochrome P450 family 1 subfamily A1
CYP1A2: Cytochrome P450 family 1 subfamily A2
PI3K/Akt: Phosphatidylinositol 3'-kinase/serine/threonine-protein kinase
MMP: Matrix metalloproteinase
CYP3A4: Cytochrome P450 family 3 subfamily A4
Aβ: β-Amyloid
GSK-3β: Glycogen synthase kinase 3β
cAMP: Cyclic AMP
DCX: Neuronal migration protein doublecortin
GFAP: Glial fibrillary acidic protein
TNF-α: Tumour necrosis factor superfamily-α
COX-2: Cyclooxygenase-2
NF-κB: Nuclear factor-kappa B
TLR4: Toll-like receptor4
VEGF: Vascular endothelial growth factor
BACE1: Beta-site amyloid precursor protein cleaving enzyme 1
CNS: Central nervous system
HMGB-1: High mobility group protein B1
ASC: Apoptosis-associated speck-like protein containing a CARD
Nrf2: Nuclear factor erythroid 2-related factor 2
AChE: Acetylcholinesterase
ChAT: Choline O-acetyltransferase
BDNF: Brain-derived neurotrophic factor
PKA: Protein kinase A
ERK: Mitogen-activated protein kinase 1/3
CaMKII: Ca2 + /calmodulin-dependent protein kinase II
iNOS: Inducible NO synthase
MDA: Monochrome display adapter
SOD: Superoxide dismutase
HO-1: Heme oxygenase 1
GSH: Glutathione
MAPK: Mitogen-activated protein kinase
IL-1β: Interleukin-1β
IL-6: Interleukin-6
TrkB: Neurotrophic tyrosine kinase receptor type 2
IL-10: Interleukin-10
PER2: Period circadian protein 2
NDUFV1: NADH dehydrogenase (ubiquinone) flavoprotein 1
JAK2/STAT3: Janus kinase 2/signal transducers 3
GRP78: Heat shock protein family A (Hsp70) member 5
NMDA: N Methyl-D-aspartate

## References

[1] Adhikari-Devkota A, Kurauchi Y, Yamada T, Katsuki H, Watanabe T, Devkota HP (2019). Anti-neuroinflammatory activities of extract and polymethoxyflavonoids from immature fruit peels of Citrus 'Hebesu'. J Food Biochem.

[2] Al RM, Nakajima A, Saigusa D, Tetsu N, Maruyama Y, Shibuya M, Yamakoshi H, Tomioka Y, Iwabuchi Y, Ohizumi Y (2009). 4'-Demethylnobiletin, a bioactive metabolite of nobiletin enhancing PKA/ERK/CREB signaling, rescues learning impairment associated with NMDA receptor antagonism via stimulation of the ERK cascade. Biochemistry-Us.

[3] Amarsanaa K, Kim HJ, Ko EA, Jo J, Jung SC (2021). Nobiletin exhibits neuroprotective effects against mitochondrial complex I inhibition via regulating apoptotic signaling. Exp Neurobiol.

[4] Asakawa T, Hiza A, Nakayama M, Inai M, Oyama D, Koide H, Shimizu K, Wakimoto T, Harada N, Tsukada H (2011). PET imaging of nobiletin based on a practical total synthesis. Chem Commun.

[5] Bi J, Zhang H, Lu J, Lei W (2016). Nobiletin ameliorates isoflurane-induced cognitive impairment via antioxidant, anti-inflammatory and anti-apoptotic effects in aging rats. Mol Med Rep.

[6] Breitinger U, Sticht H, Breitinger HG (2021). Modulation of recombinant human alpha 1 glycine receptor by flavonoids and gingerols. Biol Chem.

[7] Cao H, Chen X, Jassbi AR, Xiao J (2015). Microbial biotransformation of bioactive flavonoids. Biotechnol Adv.

[8] Chai W, Zhang J, Xiang Z, Zhang H, Mei Z, Nie H, Xu R, Zhang P (2022). Potential of nobiletin against Alzheimer's disease through inhibiting neuroinflammation. Metab Brain Dis.

[9] Cui Y, Wu J, Jung SC, Park DB, Maeng YH, Hong JY, Kim SJ, Lee SR, Kim SJ, Kim SJ (2010). Anti-neuroinflammatory activity of nobiletin on suppression of microglial activation. Biol Pharm Bull.

[10] Fujiwara H, Kimura J, Sakamoto M, Yokosuka A, Mimaki Y, Murata K, Yamaguchi K, Ohizumi Y (2014). Nobiletin, a flavone from Citrus depressa, induces gene expression and increases the protein level and activity of neprilysin in SK-N-SH cells. Can J Physiol Pharm.

[11] Fujiwara H, Kogure A, Sakamoto M, Yamakuni T, Mimaki Y, Murata K, Hitomi N, Yamaguchi K, Ohizumi Y (2011). Honeybee royal jelly and nobiletin stimulate CRE-mediated transcription in ERK-independent and -dependent fashions, respectively, in PC12D cells. J Pharmacol Sci.

[12] Ghasemi-Tarie R, Kiasalari Z, Fakour M, Khorasani M, Keshtkar S, Baluchnejadmojarad T, Roghani M (2022). Nobiletin prevents amyloid beta(1–40)-induced cognitive impairment via inhibition of neuroinflammation and oxidative/nitrosative stress. Metab Brain Dis.

[13] Gile J, Scott B, Eckle T (2018). The period 2 enhancer nobiletin as novel therapy in murine models of circadian disruption resembling delirium. Crit Care Med.

[14] Han J (2019). Chemical aspects of gut metabolism of flavonoids. Metabolites.

[15] He W, Li Y, Liu M, Yu H, Chen Q, Chen Y, Ruan J, Ding Z, Zhang Y, Wang T (2018). *Citrus aurantium* L. and its flavonoids regulate TNBS-induced inflammatory bowel disease through anti-inflammation and suppressing isolated Jejunum contraction. Int J Mol Sci.

[16] Hilel AS, Gysemans B, Lisboa M, Heymanns AC, Freiberger V, Ventura L, Magnago RF, Comim CM, Horewics V, Martins DF (2018). Dextran sulphate of sodium-induced colitis in mice: antihyperalgesic effects of ethanolic extract of Citrus reticulata and potential damage to the central nervous system. An Acad Bras Cienc.

[17] Ho SC, Kuo CT (2014). Hesperidin, nobiletin, and tangeretin are collectively responsible for the anti-neuroinflammatory capacity of tangerine peel (Citri reticulatae pericarpium). Food Chem Toxicol.

[18] Huang HJ, Chang TT, Chen HY, Chen CY (2014). Finding inhibitors of mutant superoxide dismutase-1 for amyotrophic lateral sclerosis therapy from traditional Chinese medicine. Evid-Based Compl Alt.

[19] Ihara H, Yamamoto H, Ida T, Tsutsuki H, Sakamoto T, Fujita T, Okada T, Kozaki S (2012). Inhibition of nitric oxide production and inducible nitric oxide synthase expression by a polymethoxyflavone from young fruits of Citrus unshiu in rat primary astrocytes. Biosci Biotech Bioch.

[20] Jahan S, Ansari UA, Siddiqui AJ, Iqbal D, Khan J, Banawas S, Alshehri B, Alshahrani MM, Alsagaby SA, Redhu NS (2022). Nobiletin ameliorates cellular damage and stress response and restores neuronal identity altered by sodium arsenate exposure in human iPSCs-derived hNPCs. Pharmaceuticals-Base.

[21] Jahan S, Redhu NS, Siddiqui AJ, Iqbal D, Khan J, Banawas S, Alaidarous M, Alshehri B, Mir SA, Adnan M (2022). Nobiletin as a neuroprotectant against NMDA receptors: an in silico approach. Pharmaceutics.

[22] Jeong KH, Jeon MT, Kim HD, Jung UJ, Jang MC, Chu JW, Yang SJ, Choi IY, Choi MS, Kim SR (2015). Nobiletin protects dopaminergic neurons in the 1-methyl-4-phenylpyridinium-treated rat model of Parkinson's disease. J Med Food.

[23] Kawahata I, Suzuki T, Rico EG, Kusano S, Tamura H, Mimaki Y, Yamakuni T (2017). Fermented Citrus reticulata (ponkan) fruit squeezed draff that contains a large amount of 4'-demethylnobiletin prevents MK801-induced memory impairment. J Nat Med-Tokyo.

[24] Kawahata I, Yoshida M, Sun W, Nakajima A, Lai Y, Osaka N, Matsuzaki K, Yokosuka A, Mimaki Y, Naganuma A (2013). Potent activity of nobiletin-rich Citrus reticulata peel extract to facilitate cAMP/PKA/ERK/CREB signaling associated with learning and memory in cultured hippocampal neurons: identification of the substances responsible for the pharmacological action. J Neural Transm.

[25] Kazak F, Akalin PP, Yarim GF, Baspinar N, Ozdemir O, Ates MB, Altug ME, Deveci M (2021). Protective effects of nobiletin on cisplatin induced neurotoxicity in rats. Int J Neurosci.

[26] Kim E, Nohara K, Wirianto M, Escobedo GJ, Lim JY, Morales R, Yoo SH, Chen Z (2021). Effects of the clock modulator nobiletin on circadian rhythms and pathophysiology in female mice of an Alzheimer's disease model. Biomolecules.

[27] Kimura J, Shimizu K, Kajima K, Yokosuka A, Mimaki Y, Oku N, Ohizumi Y (2018). Nobiletin reduces intracellular and extracellular beta-amyloid in iPS cell-derived Alzheimer's disease model neurons. Biol Pharm Bull.

[28] Koga N, Matsuo M, Ohta C, Haraguchi K, Matsuoka M, Kato Y, Ishii T, Yano M, Ohta H (2007). Comparative study on nobiletin metabolism with liver microsomes from rats, Guinea pigs and hamsters and rat cytochrome p450. Biol Pharm Bull.

[29] Lan HC, Li SZ, Li K, Liu EH (2021). In vitro human intestinal microbiota biotransformation of nobiletin using liquid chromatography-mass spectrometry analysis and background subtraction strategy. J Sep Sci.

[30] Lee HJ, Lee SK, Lee DR, Choi BK, Le B, Yang SH (2019). Ameliorating effect of Citrus aurantium extracts and nobiletin on beta-amyloid (1–42)-induced memory impairment in mice. Mol Med Rep.

[31] Lee JH, Amarsanaa K, Wu J, Jeon SC, Cui Y, Jung SC, Park DB, Kim SJ, Han SH, Kim HW (2018). Nobiletin attenuates neurotoxic mitochondrial calcium overload through K(+) influx and DeltaPsi(m) across mitochondrial inner membrane. Korean J Physiol Pha.

[32] Li F, Zhang K, Yu M, Chen T, Ma L, Zhang H, Jia H, Zou Z (2021). Antidepressant-like effect and phytochemical profile of supercritical CO(2) extract from Citri reticulatae pericarpium. Pharmazie.

[33] Li J, Zhou Y, Liu BB, Liu Q, Geng D, Weng LJ, Yi LT (2013). Nobiletin ameliorates the deficits in hippocampal BDNF, TrkB, and Synapsin I induced by chronic unpredictable mild stress. Evid-Based Compl Alt.

[34] Li S, Sang S, Pan MH, Lai CS, Lo CY, Yang CS, Ho CT (2007). Anti-inflammatory property of the urinary metabolites of nobiletin in mouse. Bioorg Med Chem Lett.

[35] Li S, Wang Z, Sang S, Huang MT, Ho CT (2006). Identification of nobiletin metabolites in mouse urine. Mol Nutr Food Res.

[36] Li ZR, Yang L, Zhen J, Zhao Y, Lu ZN (2018). Nobiletin protects PC12 cells from ERS-induced apoptosis in OGD/R injury via activation of the PI3K/AKT pathway. Exp Ther Med.

[37] Matsuzaki K, Miyazaki K, Sakai S, Yawo H, Nakata N, Moriguchi S, Fukunaga K, Yokosuka A, Sashida Y, Mimaki Y (2008). Nobiletin, a citrus flavonoid with neurotrophic action, augments protein kinase A-mediated phosphorylation of the AMPA receptor subunit, GluR1, and the postsynaptic receptor response to glutamate in murine hippocampus. Eur J Pharmacol.

[38] Matsuzaki K, Yamakuni T, Hashimoto M, Haque AM, Shido O, Mimaki Y, Sashida Y, Ohizumi Y (2006). Nobiletin restoring beta-amyloid-impaired CREB phosphorylation rescues memory deterioration in Alzheimer's disease model rats. Neurosci Lett.

[39] Nagase H, Omae N, Omori A, Nakagawasai O, Tadano T, Yokosuka A, Sashida Y, Mimaki Y, Yamakuni T, Ohizumi Y (2005). Nobiletin and its related flavonoids with CRE-dependent transcription-stimulating and neuritegenic activities. Biochem Bioph Res Co.

[40] Nakajima A, Aoyama Y, Shin EJ, Nam Y, Kim HC, Nagai T, Yokosuka A, Mimaki Y, Yokoi T, Ohizumi Y (2015). Nobiletin, a citrus flavonoid, improves cognitive impairment and reduces soluble Abeta levels in a triple transgenic mouse model of Alzheimer's disease (3XTg-AD). Behav Brain Res.

[41] Nakajima A, Yamakuni T, Haraguchi M, Omae N, Song SY, Kato C, Nakagawasai O, Tadano T, Yokosuka A, Mimaki Y (2007). Nobiletin, a citrus flavonoid that improves memory impairment, rescues bulbectomy-induced cholinergic neurodegeneration in mice. J Pharmacol Sci.

[42] Nakamoto A, Hirabayashi Y, Anzaki C, Nakamoto M, Shuto E, Sakai T (2021). Nobiletin suppresses the development of experimental autoimmune encephalomyelitis mediated by modulation of T helper 17 cell differentiation. J Clin Biochem Nutr.

[43] Olofinsan KA, Salau VF, Erukainure OL, Islam MS (2022). Harpephyllum caffrum fruit (wild plum) facilitates glucose uptake and modulates metabolic activities linked to neurodegeneration in isolated rat brain: an in vitro and in silico approach. J Food Biochem.

[44] Onoue S, Uchida A, Takahashi H, Seto Y, Kawabata Y, Ogawa K, Yuminoki K, Hashimoto N, Yamada S (2011). Development of high-energy amorphous solid dispersion of nanosized nobiletin, a citrus polymethoxylated flavone, with improved oral bioavailability. J Pharm Sci-Us.

[45] Onozuka H, Nakajima A, Matsuzaki K, Shin RW, Ogino K, Saigusa D, Tetsu N, Yokosuka A, Sashida Y, Mimaki Y (2008). Nobiletin, a citrus flavonoid, improves memory impairment and Abeta pathology in a transgenic mouse model of Alzheimer's disease. J Pharmacol Exp Ther.

[46] Pang Y, Yang N, Zheng Y, Zhang L, He Y, Ding W (2022). Nobiletin protects enteric nerves and ameliorates disordered bowel motility in diet-induced obese mice via increasing Trem2 expression. Biochem Bioph Res Co.

[47] Pietta PG (2000). Flavonoids as antioxidants. J Nat Prod.

[48] Qi G, Mi Y, Fan R, Li R, Liu Z, Liu X (2019). Nobiletin protects against systemic inflammation-stimulated memory impairment via MAPK and NF-kappaB signaling pathways. J Agric Food Chem.

[49] Rooprai HK, Kandanearatchi A, Maidment SL, Christidou M, Trillo-Pazos G, Dexter DT, Rucklidge GJ, Widmer W, Pilkington GJ (2001). Evaluation of the effects of swainsonine, captopril, tangeretin and nobiletin on the biological behaviour of brain tumour cells in vitro. Neuropath Appl Neurobiol.

[50] Saigusa D, Shibuya M, Jinno D, Yamakoshi H, Iwabuchi Y, Yokosuka A, Mimaki Y, Naganuma A, Ohizumi Y, Tomioka Y (2011). High-performance liquid chromatography with photodiode array detection for determination of nobiletin content in the brain and serum of mice administrated the natural compound. Anal Bioanal Chem.

[51] Sasaki K, Tateoka N, Ando H, Yoshizaki F (2005). Effect of flavones on rat brain and lung matrix metalloproteinase activity measured by film in-situ zymography. J Pharm Pharmacol.

[52] Seo TB, Jeon YA, Kim SS, Lee YJ (2021). In vitro and in vivo effects of nobiletin on DRG neurite elongation and axon growth after sciatic nerve injury. Int J Environ Res Public Health.

[53] Sharikadze N, Jojua N, Sepashvili M, Zhuravliova E, Mikeladze DG (2016). Mitochondrial target of nobiletin's action. Nat Prod Commun.

[54] Shimazu R, Anada M, Miyaguchi A, Nomi Y, Matsumoto H (2021). Evaluation of blood–brain barrier permeability of polyphenols, anthocyanins, and their metabolites. J Agr Food Chem.

[55] Singh SP, Wahajuddin TD, Patel K, Jain GK (2011). Permeability determination and pharmacokinetic study of nobiletin in rat plasma and brain by validated high-performance liquid chromatography method. Fitoterapia.

[56] Sun Z, Yang N, Jia X, Song Y, Han D, Wang X, Sun J, Li Z, Zuo Z, Guo X (2022). Nobiletin attenuates anesthesia/surgery-induced neurocognitive decline by preserving the expression of clock genes in mice. Front Neurosci-Switz.

[57] Takano K, Tabata Y, Kitao Y, Murakami R, Suzuki H, Yamada M, Iinuma M, Yoneda Y, Ogawa S, Hori O (2007). Methoxyflavones protect cells against endoplasmic reticulum stress and neurotoxin. Am J Physiol Cell Physiol.

[58] Takito J, Kimura J, Kajima K, Uozumi N, Watanabe M, Yokosuka A, Mimaki Y, Nakamura M, Ohizumi Y (2016). Nerve growth factor enhances the CRE-dependent transcriptional activity activated by nobiletin in PC12 cells. Can J Physiol Pharm.

[59] Wang CC, Kong JY, Xue CH, Zhang TT, Wang YM (2023). Antarctic krill oil exhibited synergistic effects with nobiletin and theanine on regulating ligand-specific receptor-mediated transcytosis in blood–brain barrier by inhibiting alkaline phosphatase in SAMP8 mice. Mol Nutr Food Res.

[60] Wang D, Gao F, Hu F, Wu J (2022). Nobiletin alleviates astrocyte activation and oxidative stress induced by hypoxia in vitro. Molecules.

[61] Wang H, Guo Y, Qiao Y, Zhang J, Jiang P (2020). Nobiletin ameliorates NLRP3 inflammasome-mediated inflammation through promoting autophagy via the AMPK pathway. Mol Neurobiol.

[62] Wang T, Wang F, Yu L, Li Z (2019). Nobiletin alleviates cerebral ischemic-reperfusion injury via MAPK signaling pathway. Am J Transl Res.

[63] Wang Y, Zang W, Ji S, Cao J, Sun C (2019). Three Polymethoxyflavones Purified from Ougan (Citrus reticulata Cv. Suavissima) Inhibited LPS-Induced NO Elevation in the Neuroglia BV-2 Cell Line via the JAK2/STAT3 Pathway. Nutrients.

[64] Wirianto M, Wang CY, Kim E, Koike N, Gomez-Gutierrez R, Nohara K, Escobedo GJ, Choi JM, Han C, Yagita K (2022). The clock modulator nobiletin mitigates astrogliosis-associated neuroinflammation and disease hallmarks in an Alzheimer's disease model. Faseb J.

[65] Xiong YJ, Chen DP, Lv BC, Liu FF, Wang L, Lin Y (2014). Characteristics of nobiletin-induced effects on jejunal contractility. Fitoterapia.

[66] Yabuki Y, Ohizumi Y, Yokosuka A, Mimaki Y, Fukunaga K (2014). Nobiletin treatment improves motor and cognitive deficits seen in MPTP-induced Parkinson model mice. Neuroscience.

[67] Yamamoto Y, Shioda N, Han F, Moriguchi S, Nakajima A, Yokosuka A, Mimaki Y, Sashida Y, Yamakuni T, Ohizumi Y (2009). Nobiletin improves brain ischemia-induced learning and memory deficits through stimulation of CaMKII and CREB phosphorylation. Brain Res.

[68] Yang B, Wang J, Zhang N (2018). Effect of nobiletin on experimental model of epilepsy. Transl Neurosci.

[69] Yao J, Zhou JP, Ping QN, Lu Y, Chen L (2008). Distribution of nobiletin chitosan-based microemulsions in brain following i.v. injection in mice. Int J Pharmaceut.

[70] Yarim GF, Yarim M, Sozmen M, Gokceoglu A, Ertekin A, Kabak YB, Karaca E (2022). Nobiletin attenuates inflammation via modulating proinflammatory and antiinflammatory cytokine expressions in an autoimmune encephalomyelitis mouse model. Fitoterapia.

[71] Yasuda N, Ishii T, Oyama D, Fukuta T, Agato Y, Sato A, Shimizu K, Asai T, Asakawa T, Kan T (2014). Neuroprotective effect of nobiletin on cerebral ischemia-reperfusion injury in transient middle cerebral artery-occluded rats. Brain Res.

[72] Yasuda T, Yoshimura Y, Yabuki H, Nakazawa T, Ohsawa K, Mimaki Y, Sashida Y (2003). Urinary metabolites of nobiletin orally administered to rats. Chem Pharm Bull.

[73] Yi L, Li H, Deng Q, Yuan Z (2010). Study of nobiletin binding to bovine serum albumin by capillary electrophoresis-frontal analysis and circular dichroism. Biomed Chromatogr.

[74] Yi LT, Xu HL, Feng J, Zhan X, Zhou LP, Cui CC (2011). Involvement of monoaminergic systems in the antidepressant-like effect of nobiletin. Physiol Behav.

[75] Youn K, Lee S, Jun M (2019). Discovery of nobiletin from citrus peel as a potent inhibitor of beta-amyloid peptide toxicity. Nutrients.

[76] Youn K, Yu Y, Lee J, Jeong WS, Ho CT, Jun M (2017). Polymethoxyflavones: novel beta-secretase (BACE1) inhibitors from citrus peels. Nutrients.

[77] Zhang L, Zhang X, Zhang C, Bai X, Zhang J, Zhao X, Chen L, Wang L, Zhu C, Cui L (2016). Nobiletin promotes antioxidant and anti-inflammatory responses and elicits protection against ischemic stroke in vivo. Brain Res.

[78] Zhang L, Zhao H, Zhang X, Chen L, Zhao X, Bai X, Zhang J (2013). Nobiletin protects against cerebral ischemia via activating the p-Akt, p-CREB, BDNF and Bcl-2 pathway and ameliorating BBB permeability in rat. Brain Res Bull.

[79] Zhang M, Zhang X, Zhu J, Zhao DG, Ma YY, Li D, Ho CT, Huang Q (2021). Bidirectional interaction of nobiletin and gut microbiota in mice fed with a high-fat diet. Food Funct.

[80] Zheng Y, Bu J, Yu L, Chen J, Liu H (2017). Nobiletin improves propofol-induced neuroprotection via regulating Akt/mTOR and TLR 4/NF-kappaB signaling in ischemic brain injury in rats. Biomed Pharmacother.

[81] Zhu C, Tian M, Liu N, Ma L, Lan X, Yang J, Du J, Ma H, Li Y, Zheng P (2022). Analgesic effect of nobiletin against neuropathic pain induced by the chronic constriction injury of the sciatic nerve in mice. Phytother Res.

